# Identification of positive childhood experiences with the potential to mitigate childhood unhealthy weight status in children within the context of adverse childhood experiences: a prospective cohort study

**DOI:** 10.1186/s12889-024-20727-y

**Published:** 2025-01-13

**Authors:** Brooklyn M. Mellar, Maryam Ghasemi, Pauline Gulliver, Barry Milne, Fiona Langridge, Tracey McIntosh, Christa Fouche, Boyd Swinburn, Ladan Hashemi

**Affiliations:** 1 Faculty of Medical and Health Sciences, University of Auckland, Auckland, New Zealand; 2 Intergenerational Health Research Group, Murdoch Children’s Research Institute, Melbourne, VIC Australia; 3 Faculty of Education and Social Work, University of Auckland, Auckland, New Zealand; 4 Centre of Methods and Policy Application in Social Sciences, University of Auckland, Auckland, New Zealand; 5 Wānanga o Waipapa School of Māori Studies and Pacific Studies, University of Auckland, Auckland, New Zealand; 6 Violence and Society Centre, City St George’s, University of London, London, UK

**Keywords:** Positive childhood experiences, Adverse childhood experiences, Childhood obesity, Mitigation

## Abstract

**Background:**

Despite potential protective and mitigating effects of positive childhood experiences (PCEs) on poor health outcomes, limited research has identified relevant PCEs and examined their individual and cumulative associations with weight status, or their mitigating effects on the associations between adverse childhood experiences (ACEs) and obesity in children. This study aims to develop an exploratory PCEs Index with the potential to protect against or mitigate the association between ACEs and unhealthy weight status.

**Methods:**

Data came from the Growing Up in New Zealand study. The analytic sample was restricted to those who provided obesity data at age 8 and one child per mother, resulting in a sample of 4,895 children. Nine individual ACEs and their cumulative scores, a newly developed PCEs index consisting of six individual PCEs and (their) cumulative scores, and an overweight/obesity variable were included in the analyses.

**Results:**

By age eight, experience of at least 3 PCEs was reported by 72.1% of the sample. However, the experience of the highest number of PCEs (5–6) was only reported by 23% of the sample. Four out of six assessed PCEs were associated with decreased likelihood of overweight/obesity. A dose-response effect was observed where experience of three or more PCEs was associated with decreased odds for obesity (AORs decreased from 0.77 for 3 PCEs to 0.54 for 5–6 PCEs). No consistent mitigating effects were found for individual PCEs; however interactions were found between reporting at least four of the six PCEs, experience of cumulative ACEs, and reduced odds for overweight/obesity at age 8.

**Conclusions:**

A critical number of PCEs may be required to mitigate the detrimental impacts of ACEs on weight status among children. These findings reinforce the need to consider a constellation of strength-focused ecological domains to alleviate the burden of childhood obesity, particularly for children exposed to multiple adversities.

**Supplementary Information:**

The online version contains supplementary material available at 10.1186/s12889-024-20727-y.

## Introduction

Overweight and obesity in childhood have long-term impacts on health and wellbeing [1–4], and rates are increasing internationally [5]. In light of unsuccessful child obesity reduction efforts, the contributions of a broader range of social, emotional, and environmental contexts of child development must be examined to gain a more complete understanding of possible risk and protective factors for unhealthy weight in children [6, 7].

International research has shown that individuals impacted by Adverse Childhood Experiences (ACEs) are at greater risk of developing obesity [7–9], in addition to a range of poor physical and mental health outcomes across the lifespan [10–13]. ACEs include indicators of child abuse and family dysfunctions (e.g., mental illness, substance abuse, incarceration, parental separation or death, and intimate partner violence) [10, 12]. Most research linking ACEs and obesity has measured this relationship in adults, but there is limited information about this risk in children [8]. In response to critiques that ACEs research overemphasizes risks in lieu of protective factors [14, 15], research has recently begun to identify positive childhood experiences (PCEs) that may promote childhood resilience, healthy development, and counter the damaging effects of adversity [15–19]. PCEs encompass a range of domains including family and parenting environment, healthcare services and education, peer relationships, cultural and community connectedness, and neighborhood environment [20, 21].

PCEs remain relatively underexplored compared with ACEs research [22]; for example, most existing data has relied on small or non-representative and standardized PCE measurement tools are underdeveloped or designed for retrospective assessment of PCEs during adulthood [20, 21, 23]. Further, prior research has focused on the protective role of PCEs using a limited range of PCEs [20, 24], or independently examined individual PCEs [17]. Scholars have emphasized the need for ecological models of childhood experiences that draw on a constellation of environmental factors [17]. This aligns with theoretical frameworks such as Bronfenbrenner’s Ecological Systems Theory [25], which posits that child development is influenced by a range of interconnected systems, within which ACEs and PCEs coexist and shape outcomes [20, 21].

Resilience has been posited as a protective mechanism for obesity prevention in childhood [8]. Understanding what differentiates children who exhibit resilience is critical for improving the effectiveness of health-related prevention and intervention efforts for children exposed to adversity. Research shows that resilience-building traits, (such as self-esteem, emotional regulation, and prosocial skills), equip children to manage stress, foster positive social interactions, and maintain a sense of self-worth [26–28]. PCEs contribute to the development of resilience-building traits [29], which may potentially disrupt the theorized biological pathways linking ACEs to poor health and well-being [20, 30–34]. However, limited research has examined the association between PCEs and weight status or their mitigating effects in ACEs-obesity association in children and adolescents [24, 35]. Further research is required to understand these connections and to identify crucial intervention points for addressing physical and mental health challenges associated with ACEs [17]. The present study aims to address these gaps by developing an exploratory PCE Index to further understanding of the relationship between PCEs and unhealthy weight status in a prospective cohort of New Zealand (NZ) children, with potential to mitigate the association between ACEs and unhealthy weight status. This study focuses on identifying PCEs that may be modifiable through public health interventions or policy initiatives, which, unlike non-modifiable factors such as genetic predispositions, are actionable targets for child obesity reduction efforts.

## Methods

### Population and data

Participants were members of Growing Up in NZ (GUiNZ), a contemporary NZ-based prospective cohort study, for which methodological details can be found elsewhere [36]. The study antenatally recruited 6853 children born between 2009 and 2010; characteristics of the original cohort aligned with the national birth cohort during 2007-2010 [36]. Data collection waves (DCWs) used face-to-face and phone interviews to gather information on various aspects of the children’s development and family environment. Ethics approval was granted by the NZ Ministry of Health Northern Y Regional Ethics Committee, and all enrolled parents provided informed consent.

Follow-up procedures included regular data collection points from pregnancy through various stages of the children’s development. Major DCWs occurred antenatally, at birth, at 6 weeks, at 9 months, at 2 years, at 4.5 years, and at 8 years, up to the time of this study.

The current study was restricted to one child per participating mother and to those who provided obesity data during the age 8 DCW (2017–2019), resulting in an analytic sample of 4,895 children. Compared to children recruited at baseline, children lost to attrition by DCW8 were more likely to be in lower socioeconomic groups, of an ethnicity other than European, have younger mothers, and present BMI in the range of overweight/obesity at age 4.5 years (Supplementary Table 3).

Reporting of this analysis follows the Strengthening the Reporting of Observational Studies in Epidemiology (STROBE) guidelines [37].

### Key measures

#### ACEs

A literature review was conducted to compile a comprehensive list of commonly studied ACEs. All available GUiNZ datasets and instruments (from antenatal data collection up to age 8) were systematically reviewed to identify proxies for commonly investigated ACEs. To capture a wide range of adversities and reduce bias associated with parental reporting, we included both child and parent reports and utilised standardised questionnaires across eight waves of GUiNZ data.

Specifically, we constructed dichotomous indicators of exposure for nine ACEs: child exposure to emotional abuse, physical abuse, parental substance (alcohol/drug) abuse, parental mental illness, parental incarceration, parental separation/divorce, intimate partner violence (IPV) against the mother, maternal experience of ethnic discrimination, and peer bullying. Each ACE was identified through multiple questions, with predetermined criteria applied to synthesise responses; an ACE was considered present if it was reported in one or more questions, regardless of any inconsistencies across responses. Due to the absence of direct data on children’s personal experiences of ethnic discrimination, we used the mother’s exposure to ethnic discrimination as a proxy for this ACE.

An ACE score (0, 1, 2, 3, or 4 + ACEs) was generated to reflect the cumulative exposure to adversities. ACE measures are detailed in Supplementary Table 1.

#### Overweight/obesity

Body mass was measured by interviewers collecting child’s weight and height measurements during the DCW at age 8. BMI-for-age z scores were derived using World Health Organization 2006 child growth chart standards and binarized into normal or underweight versus overweight or obesity. Combined overweight/obesity was used to ensure sufficient data density for interactions and enable comparability with prior studies [24]. BMI categories were utilized to enhance the clarity of the findings for non-specialists (such as policymakers, educators, and healthcare providers), supporting the translation of them into interventions and policy recommendations.

#### PCEs

Given the plethora of instruments and variables collected on potential PCEs in GUiNZ, a post-hoc approach was employed for PCEs selection to explore the under-researched field of PCEs and child weight. PCEs were based on the Healthy Outcomes Positive Experiences (HOPE) Framework developed by Sege and Browne, as it provided a well-supported conceptual basis for development of a PCE Index using available GUiNZ data [15, 38]. HOPE comprises four broad categories for PCEs to align with: (1) being in nurturing, supportive relationships, (2) living, developing, playing, and learning in safe, stable, protective, and equitable environments, (3) having opportunities for constructive social engagement and connectedness, (4) learning social and emotional competencies [15].

PCE measures which focused on the presence of assets (rather than absence of adversity) were mapped to three HOPE categories, as the fourth domain (learning social and emotional competencies) may be an indicator of outcomes [22]. PCEs collected prior to DCW8 were prioritized to enable temporal sequencing by comparison with age 8 outcomes.

For refinement, PCEs were selected if they were achievable and modifiable, and met at least one of the following criteria:

- Associated with reduced odds of reporting overweight/obesity at age 8 (after sociodemographic adjustment), to test predictive validity of the PCE index for overweight/obesity;
- Interacted with reduced odds of reporting overweight/obesity at age 8 (after sociodemographic adjustment) for at least one ACEs score;
- Closely bordered decreased odds for overweight/obesity at age 8, if supported by published literature.

Six PCEs were selected: parents in a committed relationship, mother interacted well with child, mother involved in social groups, child engaged in experiences and activities, child lived in a house with routines and rules, and child attended effective early childhood education. PCE measures were transformed into binary variables (Yes/No) set around the 55th percentile of the sample responses, to capture slightly better than average responses. PCE measures are detailed in Table 1.

**Table 1:** Measures used to define positive childhood experiences using data from the Growing up in New Zealand Study

PCE	HOPE Category	Data Collection Wave (DCW)	Questions & Response Options	Threshold/Scoring	Notes
Mother in committed relationship	(2) Living in a safe & stable environment	1, M	“I want to grow old with my partner” “When I imagine what my life will be like in the future, I always see my partner standing next to me” Likert Scale: (1) Strongly disagree, (2) disagree, (3) not sure, (4) agree, (5) strongly agree	Median/55th percentile ‘Strongly agree’ to both questions (score: 10), versus low personal commitment or having no partner.	Questions pertaining to moral and structural commitment were excluded as the three measured types of commitment can be differently associated with outcomes, and have low correlation with each other [63].
Mother interacted well with child	(1) Being in nurturing, supportive relationships	5, O	Children and their mothers undertook a set task (creation of a party invitation), and via observed measures interviewers’ assessed the quality of how the mother interacted: a) promoted the child’s learning (used who/what/where/how/ why questions and engaged in print talk e.g., “How do you draw a b”, b) provided support and sensitive assistance (mother and child completed task together, versus either dominating the task), and c) gave warmth and encouragement (instances of praise e.g., “Nice one!”.	Median/55th percentile Derived from continuous score from four measures.	A measure on child’s focus during the task was excluded to center the mother’s role in the interaction and to ensure that the measure was not inadvertently biased against children with behavioral issues. The task has been used among a broad range of cultures and socioeconomic backgrounds [64, 65]. Further detail on this measure is provided elsewhere [66].
Mother involved in social groups	(3) Having opportunities for constructive social engagement & connectedness.	1, M	“Do you belong to any of these social networks, groups or organizations”: 9 options: sports club; church or spiritual group; hobby or interest group; community/voluntary group; education group; parent and baby; ethnic/cultural groups; Marae; family/whanau; online group; people from work/school; other	Median/55th percentile At least 2 groups	As in the HOPE principles, children’s PCEs also encompass their parents’ positive experiences; mother’s social engagement and participation in social and community groups has been posited to provide a link between family and community via parental support and social integration, and influence child perception of and participation in organized activities [67].
Child engaged in experiences and activities	(2) Developing, playing & learning in safe & stable environments (3) Having opportunities for constructive social engagement & connectedness	2, M	“Which of the these activities [child] has done or places [child] has been at any time since [he/she] was born”: 37 options: library; park; beach; Santa parade; cinema; church, temple, mosque; art gallery; swimming lessons; music groups; playgroup; zoo; aquarium; museum; Diwali; lantern festival; Matariki; White Sunday; flea market; farmers market; Pasifika festival; Polyfest; Marae event; agricultural field days; mustering; nature walks; coffee groups; organised physical activity; picnics; watching sports; community galas/fairs; overseas travel; transport experience; aquatic/outdoor activities; concert, play, live show; other	Median/55th percentile At least 13 activities	
Child lived in a home with routines and rules	(2) Living, developing, playing, & learning in safe & stable environments	2 & 4, M	a) “Are there rules about: what TV programs your child can watch? How many hours of TV, videos, and DVDs your child can watch? When your child watches TV?” “How often do you make sure your child follows the rules about TV use?” All of the time, most of the time, half of the time, less than half of the time, never. b) “Does [name] go to bed at a similar time each night?” Never, sometimes, usually, always. c) “How many days a week does your family including [name] usually sit together to eat any main meal? Includes occasions when not all family members are present.”	If all three thresholds were met: a) Has TV rules or no TV, enforced at least most of time (reported by 53.6% of sample) b) Bedtime usually or always consistent (92.5%) c) At least 6 days per week (81.6%)	Home routines constitute part of having a safe and stable home, as well as conferring health benefits for sufficient sleep and non-sedentary playtime. Similar iterations of this PCE have been explored in various studies [17, 21, 62, 68].
Mother was satisfied with early childhood education (ECE)	(1) Being in nurturing, supportive relationships (2) Developing, playing & learning in safe & stable environments (3) Having opportunities for constructive social engagement & connectedness	5, M	“How satisfied are you with the effect that this early childhood education or care arrangement has had on your child’s:” 10 domains: independence; social skills (playing, joining in, relationships with others); development of language & communication; development of cultural awareness &/or belonging; pre-writing/writing skills; pre-reading/reading skills; skills with numbers; physical or motor skills; interest in music or singing; interest in learning & exploring. Likert scale: (1) very satisfied, (2) satisfied, (3) neither satisfied nor dissatisfied, (4) dissatisfied, (5) very dissatisfied	55th percentile Derived from continuous score from 10 Likert scales. Those who did not attend ECE were marked as not having this PCE.	Mother’s report of satisfaction of ECE effect on child can be considered a proxy for the quality of ECE. No involvement in ECE may limit children from opportunities to learn in safe environments, especially for those who have experienced ACEs. It has been previously contended that children’s involvement in quality childcare can have a protective effect for the impacts of ACEs [69, 70].

Note. DCW number corresponds to child’s age. M = Mother; O = Observed by interviewer. Median/55th percentile indicates that the median score and score at the 55th percentile were the same

PCE variables were binarized to improve result interpretability, particularly in identifying the number of PCEs needed to mitigate ACE effects. A cumulative PCE score was calculated and grouped (0–1, 2, 3, 4, and 5–6 PCEs). Given the small sample size for each cell, this enabled us to ensure adequate statistical power to detect meaningful associations and interactions (sliced up by overweight/obesity, ACEs score, and PCEs score). Four different groups of binarized PCEs scores were tested to find the optimal number of PCEs with the potential to mitigate the impact of ACEs on the outcome variable (0–1 vs. 2–6, 0–2 vs. 3–6, 0–3 vs. 4–6, 0–4 vs. 5–6).

Certain PCEs from later GUiNZ waves were omitted from our list due to potential social desirability biases or insufficient predictive validity with weight status. While the ACEs list includes adversities up to age 8, the final list of PCEs encompasses positive experiences in the child’s life up to age 5, in attempt to capture early exposure to resilience-building factors that may precede experience of ACEs.

### Covariates

Sociodemographic variables were used to explore the prevalence rates of ACEs scores, individual PCEs and PCE scores, and the overweight/obesity indicator among sub-populations and as potential confounders in multivariable analyses. Demographic characteristics of the child were child’s sex and ethnicity. Socioeconomic status measures included food insecurity and area deprivation level [39]. 

### Analytic methods

Statistical analyses were performed using Stata 15 [40]. Prevalence rates for all variables were computed, and bivariate associations between sociodemographic characteristics and ACEs scores, individual PCEs, PCE scores, and overweight/obesity were evaluated using χ^2^ tests (Tables 2 and 3).

**Table 2:** Sociodemographic distribution of the GUiNZ sample, ACEs scores, and overweight/obesity, and prevalence of overweight/obesity by ACEs scores

	Total ^a^	ACEs score					Overweight/obesity (BMI)
		0	1	2	3	4+	
	4,895 (100)	640 (12.9)	1355 (27.4)	1334 (27.0)	828 (16.7)	789 (16.0)	1682 (34.36)
**Gender**							
Boy	2,522 (51.5)	303 (11.9)	689 (27.1)	693 (27.2)	432 (16.9)	433 (17.0)	895 (35.49)
Girl	2,373 (48.5)	337 (14.1)	666 (27.8)	641 (26.8)	396 (16.5)	356 (14.9)	787(33.16)
χ^2^ (p-value)		8.52 (0.07)					2.93 (0.087)
**Child’s prioritised ethnicity** ^b^							
Māori	1,099 (22.8)	85 (7.7)	199 (17.9)	298 (26.8)	216 (19.5)	313 (28.2)	495 (45.04)
Pacific	524 (10.9)	27 (5.0)	82 (15.3)	126 (23.5)	131 (24.4)	171 (31.8)	358 (68.32)
Asian	695 (14.4)	75 (10.7)	175 (24.9)	219 (31.1)	130 (18.5)	105 (14.9)	181 (26.04)
MELAA	105 (2.2)	< 10^f^ (8.6)	35 (33.3)	35 (33.3)	18 (17.1)	< 10^f^ (7.6)	29 (27.62)
European	2,406 (49.8)	431 (17.8)	853 (35.3)	638 (26.4)	318 (13.2)	179 (7.4)	595 (24.73)
χ^2^ (p-value)		**579.85 (0.001)**					**446.06 (0.001)**
**Experienced food insecurity** ^**c**^							
No	3,689 (80.9)	543 (14.62)	1134 (30.5)	1053 (28.5)	566 (15.2)	418 (11.3)	1,116 (30.25)
Yes	871 (19.1)	62 (7.0)	141 (15.9)	189 (21.3)	193 (21.8)	301 (33.9)	423 (48.56)
χ^2^ (p-value)		**354.59 (0.001)**					**105.68 (0.001)**
**Area deprivation level (NZDep)** ^**d**^							
Least	1,745 (35.8)	308 (17.5)	610 (34.7)	491 (27.9)	221 (12.6)	128 (7.3)	454 (26.02)
Moderate	1,841 (37.8)	242 (13.1)	521 (28.1)	517 (27.9)	320 (17.3)	256 (13.8)	568 (30.85)
Most	1,283 (26.4)	86 (6.6)	218 (16.7)	317 (24.3)	280 (21.5)	403 (30.9)	650 (50.66)
χ^2^ (p-value)		**468.64 (0.001)**					**215.14 (0.001)**
**Overweight/obesity** ^**e**^		141 (22.2)	386 (28.7)	437 (33.0)	327 (40.0)	391 (50.5)	**-**
χ^2^ (p-value)		**162.76 (0.001)**					

Note. ^a^ The total sample used in the present study was restricted to those who had obesity data at DCW8

^b^ Ethnicity (DCW4) was externally prioritized based on NZ Ministry of Health protocol to the categories: Māori (NZ indigenous peoples), Pacific peoples, Asian, Middle Eastern/Latin American/African (MELAA), and NZ European/New Zealander/Other (‘European’). European and New Zealanders were combined as the majority of those who identify as New Zealanders are European [71]

^c^ Food security was identified if the mother confirmed that they could “always” afford to eat properly. Responses of “sometimes” or “never” were recorded as indicating food insecurity /(DCW8)

^d^ 2013 Index of Deprivation [39], categorized into “Least deprived (NZDep 1–3)”, “Moderately deprived (NZDep 4–7)”, and “Most deprived (NZDep 8–10)”

^e^ For prevalence of overweight/obesity by each ACEs score, column percentages are presented

^f^ Cells have fewer than 10 counts. Exact numbers not reported to protect the anonymity of participants

The bold font indicates significant p-value at *p* < .05

**Table 3:** Distribution of individual PCEs and PCEs scores in the GUiNZ sample (*n* = 4,895) by sociodemographic characteristics and ACEs scores

	Individual PCEs, n(%)						PCEs score, n(%)				
	Mother in committed relationship	Mother interacted well with child	Mother involved in social groups	Child engaged in experiences	Child lived in home with rules & routine	Mother was satisfied with ECE	0–1 PCEs	2 PCEs	3 PCEs	4 PCEs	5–6 PCEs
	3,335 (69.6)	2,800 (62.0)	3,425 (71.4)	2,599 (53.8)	1,981 (40.6)	2,359 (50.2)	534 (10.9)	831 (17.0)	1,166 (23.8)	1,237 (25.3)	1,125 (23.0)
**Gender**											
Boy	1,713 (69.5)	1,357 (59.5)	1,801 (73.0)	1,373 (55.2)	1,011 (40.2)	1,157 (49.1)	286 (11.3)	441 (17.5)	590 (23.4)	646 (25.6)	558 (22.1)
Girl	1,622 (69.7)	1,422 (64.8)	1,624 (69.7)	1,226 (52.4)	970 (41.1)	1,202 (52.9)	248 (10.5)	390 (16.4)	576 (24.3)	591 (24.9)	567 (23.9)
χ^2^ (p-value)	0.03 (0.875)	13.6 (**0.001**)	6.3 (**0.012**)	3.9 (**0.048**)	0.4 (0.514)	13.3 (**0.001**)	4.0 (0.408)				
**Child’s prioritised ethnicity**											
Māori	644 (59.7)	615 (60.1)	751 (69.7)	621 (57.3)	379 (34.6)	547 (51.4)	126 (11.5)	206 (18.7)	279 (25.4)	284 (25.8)	204 (18.6)
Pacific	304 (60.7)	267 (56.7)	352 (70.3)	220 (43.3)	158 (30.4)	236 (48.1)	89 (16.9)	120 (22.9)	133 (25.4)	104 (19.8)	78 (14.9)
Asian	447 (66.1)	292 (49.0)	407 (60.1)	230 (34.0)	229 (33.0)	265 (39.1)	150 (21.6)	162 (23.3)	185 (26.6)	123 (17.7)	75 (10.8)
MELAA	75 (74.3)	57 (63.3)	74 (73.3)	61 (58.1)	43 (40.9)	47 (39.1)	< 10 (6.7)	23 (21.9)	20 (19.0)	37 (35.2)	18 (17.1)
European	1,837 (77.1)	1,547 (67.5)	1,813 (76.0)	1,449 (60.4)	1,160 (48.2)	1,264 (53.4)	124 (5.1)	302 (12.5)	545 (22.6)	686 (28.5)	749 (31.1)
χ^2^ (p-value)	136.8 (**0.001**)	79.3 (**0.001**)	69.7 (**0.001**)	178.2 (**0.001**)	113.6 (**0.001)**	45.3 (**0.001**)	412.8 **(0.001)**				
**Experienced food insecurity**											
No	2,679 (73.9)	2,202 (64.4)	2,680 (73.9)	2,101 (57.5)	1,617 (43.9)	1,849 (51.7)	279 (7.6)	552 (15.0)	892 (24.2)	995 (27.0)	969 (26.3)
Yes	448 (53.0)	436 (55.2)	544 (64.2)	366 (43.1)	269 (31.1)	379 (46.2)	184 (21.1)	183 (21.0)	205 (23.5)	190 (21.8)	109 (12.5)
χ^2^ (p-value)	142.6 (**0.001**)	23.4 (**0.001**)	31.7 (**0.001**)	57.7 (**0.001**)	47.8 (**0.001**)	8.3 (**0.004**)	207.3 **(0.001)**				
**Area deprivation level (NZDep)**											
Least	1,328 (76.9)	1,053 (65.0)	1,289 (74.7)	1,076 (62.2)	801 (46.0)	889 (52.0)	94 (5.4)	254 (14.6)	386 (22.1)	512 (29.3)	499 (28.6)
Moderate	1,295 (71.6)	1,091 (63.7)	1,296 (71.7)	988 (54.3)	767 (41.7)	886 (49.9)	184 (10.0)	286 (15.5)	458 (24.9)	467 (25.4)	444 (24.1)
Most	692 (56.0)	624 (55.5)	827 (66.9)	517 (41.4)	403 (31.6)	574 (48.3)	251 (19.6)	288 (24.5)	318 (24.8)	246 (19.2)	180 (14.0)
χ^2^ (p-value)	154.6 (**0.001**)	28.9 (**0.001**)	21.7 (**0.001**)	126.7 (**0.001**)	64.9 (**0.001**)	4.0 (0.134)	272.9 **(0.001)**				
**ACEs Score**											
0 ACEs	531 (85.4)	391 (67.3)	468 (75.2)	371 (59.1)	319 (50.5)	323 (52.9)	30 (4.7)	85 (13.4)	133 (21.0)	179 (28.3)	206 (32.5)
1 ACE	1,038 (78.1)	795 (64.6)	1,014 (76.3)	782 (58.8)	629 (46.9)	671 (51.1)	82 (6.1)	179 (13.3)	326 (24.3)	377 (28.1)	379 (28.2)
2 ACEs	956 (74.0)	764 (62.1)	964 (74.5)	725 (55.3)	549 (41.5)	668 (52.7)	124 (9.4)	197 (14.9)	324 (24.4)	353 (26.6)	328 (24.7)
3 ACEs	470 (59.4)	426 (58.4)	518 (65.5)	387 (48.2)	266 (32.7)	370 (47.4)	132 (16.2)	177 (21.7)	199 (24.4)	181 (22.2)	128 (15.7)
4 + ACEs	340 (44.7)	403 (57.2)	461 (60.7)	334 (44.3)	218 (28.4)	327 (44.9)	166 (21.5)	24.9 (19.3)	184 (23.8)	147 (19.0)	84 (10.9)
χ2 (p-value)	390.9 (**0.001**)	21.3 (**0.001**)	82.7 (**0.001**)	59.0 (**0.001**)	116.8 (**0.001**)	16.0 (**0.003**)	347.6 **(0.001)**				

Associations between individual PCEs and PCEs scores and overweight/obesity were assessed using logistic regression analyses, presenting odds ratios unadjusted and adjusted for sociodemographic characteristics (Table 4). Associations between ACEs scores and overweight/obesity were assessed using logistic regression analyses and are reported as unadjusted and adjusted odds ratios in Table 5.

**Table 4:** Prevalence of overweight/obesity by individual PCEs and PCEs scores, and odds ratios for associations between individual PCEs, PCEs scores and overweight/obesity

	Overweight/Obesity (*n* = 1,682)		
	n(%)	OR [95% CI]	AOR [95% CI]
**Individual PCE**			
Mother in a committed relationship (*n* = 3,335)	1054 (31.6)	**0.70 [0.61–0.79]**	**0.83 [0.72–0.96]**
Mother interacted well with child (*n* = 2,800)	880 (31.7)	**0.77 [0.67–0.87]**	**0.82 [0.72–0.95]**
Mother involved in social groups (*n* = 3,425)	1133 (33.1)	**0.86 [0.75–0.98]**	0.87 [0.75–1.01]
Child engaged in experiences (*n* = 2,599)	823 (31.7)	**0.79 [0.70–0.89]**	**0.86 [0.75–0.98]**
Child lived in a home with routines and rules (*n* = 1,981)	572 (28.9)	**0.66 [0.58–0.75]**	**0.75 [0.66–0.86]**
Mother was satisfied with ECE (*n* = 2,359)	814 (34.5)	1.04 [0.92–1.17]	1.04 [0.91–1.19]
**PCE Scores**			
0–1 PCEs (*n* = 534)	242 (45.3)	**Ref.**	**Ref.**
2 PCEs (*n* = 831)	313 (37.7)	**0.73 [0.58–0.91]**	**0.75 [0.58–0.97]**
3 PCEs (*n* = 1,166)	420 (36.0)	**0.68 [0.55–0.84]**	**0.77 [0.60–0.98]**
4 PCEs (*n* = 1,237)	410 (33.1)	**0.60 [0.49–0.74]**	**0.69 [0.54–0.88]**
5–6 PCEs (*n* = 1,125)	297 (26.4)	**0.43 [0.35–0.54]**	**0.54 [0.42–0.69]**

AOR: Odd ratios adjusted for child’s gender, food insecurity, child’s ethnicity

The bold font indicates significant OR/AOR at *p* < .05

**Table 5:** Multivariable models for odds of overweight/obesity by PCEs, ACEs scores, and PCEs/ACEs interaction terms

		Overweight/obesity AOR^a^ [95%CI]			
	***Model 1*** ^***b***^	***Model 2*** ^***c***^ **0–1 (ref.) vs. 2–6 PCEs**	***Model 3*** **0–2 (ref.) vs. 3–6 PCEs**	***Model 4*** **0–3 (ref.) vs. 4–6 PCEs**	***Model 5*** **0–4 (ref.) vs. 5–6 PCEs**
Adjusted for SES covariates & unadjusted for ACEs	**-**	**0.60 [0.50–0.71]**	**0.69 [0.60–0.78]**	**0.68 [0.61–0.77]**	**0.62 [0.53–0.72]**
Adjusted for SES covariates & ACEs score	**-**	**0.75 [0.60–0.94]**	0.87 [0.75–1.01]	**0.82 [0.72–0.94]**	**0.75 [0.64–0.89]**
**ACE Index (Ref = 0)**					
* 1 ACE*	**1.45 [1.14–1.84]**	**1.44 [1.14–1.83]**	**1.45 [1.14–1.83]**	**1.43 [1.13–1.82]**	**1.43 [1.12–1.81]**
* 2 ACEs*	**1.51 [1.19–1.92]**	**1.50 [1.18–1.90]**	**1.50 [1.18–1.90]**	**1.49 [1.17–1.89]**	**1.48 [1.17–1.88]**
* 3 ACEs*	**1.84 [1.43–2.38]**	**1.80 [1.39–2.33]**	**1.80 [1.39–2.34]**	**1.77 [1.37–2.30]**	**1.77 [1.37–2.29]**
* 4 + ACEs*	**2.30 [1.77-3.00]**	**2.23 [1.71–2.90]**	**2.23 [1.71–2.91]**	**2.20 [1.68–2.87]**	**2.20 [1.68–2.86]**
**Interaction: PCEs x ACEs**					
* PCEs x 1 ACE*		1.12 [0.37–3.35]	0.97 [0.52–1.82]	**0.60 [0.36–0.99]**	**0.60 [0.36–0.99]**
* PCEs x 2 ACEs*		1.66 [0.57–4.82]	1.04 [0.56–1.92]	**0.57 [0.35–0.95]**	**0.59 [0.35–0.98]**
* PCEs x 3 ACEs*		1.47 [0.51–4.25]	1.13 [0.60–2.12]	0.62 [0.36–1.05]	0.71 [0.39–1.30]
* PCEs x 4 + ACEs*		1.13 [0.40–3.22]	0.89 [0.48–1.67]	**0.50 [0.29–0.86]**	**0.36 [0.18–0.70]**

Note. ^a^ SES covariates were child’s prioritized ethnicity (DCW4), child’s gender (DCW0), food insecurity (DCW8)

^b^ Model 1 is unadjusted for PCEs

^c^ Models 2–5 are adjusted for SES covariates, ACEs, and PCE thresholds

The bold font indicates significant OR/AOR at *p* < .05

For assessing the combined effect of cumulative PCEs and ACEs, we used a series of binary PCE scores to ensure adequate statistical power to detect meaningful interactions given the small cell sizes (sliced by overweight/obesity, ACEs score, and PCEs score). We tested four different binary PCEs scores to find the optimal number of PCEs for mitigating the impact of ACEs on overweight/obesity (0–1 vs. 2–6, 0–2 vs. 3–6, 0–3 vs. 4–6, 0–4 vs. 5–6). Separate multivariable logistic regression analyses were conducted to evaluate associations between cumulative PCEs scores and overweight/obesity while adjusting for sociodemographic characteristics, and also unadjusted and adjusted for ACEs scores (Table 5). Multivariable logistic regressions were used with interaction terms to identify changes between overweight/obesity for different PCEs and ACEs scores (Table 5).

All multivariable models were adjusted for child’s sex, child’s ethnicity, and food insecurity. We did not adjust for area-level deprivation to avoid multicollinearity between sociodemographic variables. Odds ratios are presented with 95% confidence intervals throughout, and statistical significance was set at *p* < .05.

## Results

Just over half of the study population were boys (51.5%), and half were European (49.8%). Māori children comprised 22.8% of the sample, followed by Asian (14.4%), and Pacific children (10.9%), 19.1% of the sample were identified as food insecure at DCW8 (Table 3).

ACEs exposure was prevalent in the sample: around 54% reported 1 or 2 ACEs and 32% reported at least 3 ACEs. Higher ACEs were more prevalent among Māori or Pacific children, and those living in the most deprived areas and in food insecure households (Table 3).

Overall, 34.4% of the sample’s BMI z-score placed them in the overweight/obesity category at age 8. Overweight/obesity was more prevalent among Pacific and Māori children, and those living in food insecure households or higher deprivation areas (Table 3). A trend was observed between ACEs scores and overweight/obesity, with higher ACEs scores associated with increased odds of overweight/obesity. For example, those who experienced 1 ACE were 1.45 [1.14–1.84] times more likely to present overweight/obesity at age 8, compared with those who reported no ACEs (Model 1, Table 5), the odds ratio increased to 2.30 [1.77-3.00] times for those who experienced 4 + ACEs.

Mother involved in social groups was the most prevalent PCE reported by 71.4% of the sample, followed by mother in a committed relationship (69.6%). Living in a household with routines and rules was the least prevalent PCE reported by (40.6%) of the sample. Some sociodemographic differences were observed across individual PCEs. For example, boys were more likely to have a mother involved in social groups and they were more likely to be engaged in experiences and activities than girls, whereas girls were more likely to have mothers who were satisfied with their ECE and who interacted well with them. PCEs were less prevalent among Asian children, those living in the most deprived areas, and those living in food insecure households (Table 3).

Regarding cumulative scores, experience of at least 3 PCEs was reported by 72.1% of the sample. However, around one in ten (10.9%) experienced zero or only one PCE, and the 5–6 PCEs was only reported by 23% of the sample. The prevalence of multiple PCEs varied across sociodemographic subgroups and showed similar patterns to individual PCEs. In the context of ACEs, the proportion of those reporting each individual PCE decreased as the number of ACEs increased. A similar pattern was observed for cumulative PCEs where, higher ACEs scores were accompanied by lower PCEs scores, and lower ACEs scores were accompanied by higher PCEs scores (Table 3).

Table 4 demonstrates the predictive validity of protective effects for each individual PCE on overweight/obesity at age 8; four individual PCE were associated with decreased odds of overweight/obesity. In terms of cumulative impact, a dose-response association was observed for crude associations, with additional PCEs conferring lower odds of overweight/obesity. After sociodemographic adjustment, a stepwise association was not clear for less than 3 PCEs but was maintained from 3 PCEs (AOR 0.77 [0.60–0.98]), 4 PCEs (AOR 0.69 [0.54–0.88]), to 5–6 PCEs (AOR 0.54 [0.42–0.69]).

We did not find consistent mitigating effects for individual PCEs (Supplementary Table 2). After controlling for ACEs scores and sociodemographic characteristics, associations between cumulative PCEs with overweight/obesity slightly attenuated (Table 5). Interactions were found between reporting at least four of the six PCEs, experience of 1 ACE, 2 ACE or 4 + ACEs and reduced likelihood for overweight/obesity at age 8. That is, children who experienced 4 + PCEs had lower odds of overweight/obesity at all levels of ACEs (except for 3 ACEs) than children who experienced less than 4 PCEs (Table 5).

The mitigating effect was stronger for 5–6 PCEs compared to 4–6 PCEs. Notably, among children with 4 + ACEs, those with 5–6 PCEs had 64% lower odds of overweight/obesity (AOR 0.36 [0.18–0.70]), while those with 4–6 PCEs had 50% lower odds of overweight/obesity (AOR 0.50 [0.29–0.86]). Interestingly, within the group of children with 4–6 PCEs, those with 4 + ACEs experienced lower odds of overweight/obesity than those with 1 or 2 ACEs (Table 5).

## Discussion

The PCEs Index developed in this study supported the hypothesis that PCEs may mitigate the association between ACEs and child weight at age 8 years. Our findings can inform future development of PCEs Indices for childhood obesity and other health outcomes, including further validation of tools for appropriate PCEs measurement.

Using data from a large cohort of NZ children, this study found that high counts of ACEs and PCEs were prevalent among the GUiNZ cohort with almost one in three experiencing 3 + ACEs and one in four experiencing 5–6 PCEs. However, there were observable inequities; children living in financially disadvantaged households experienced a higher number of ACEs and the lowest prevalence of experiencing all individual PCEs and lower PCEs scores. The prevalence of ACEs found in this study is substantially higher than those reported in retrospective cross-sectional studies utilizing standardized ACEs questionnaires [12, 41], but is in the range of those reported in international prospective ACEs studies [42]. Comparison of PCE prevalence rates found in other studies is difficult due to methodological inconsistencies, such as varying PCE definitions (including cumulative versus individual PCEs) [29] and sample heterogeneity (notably retrospective PCEs assessment in adults) [14].

In line with previous studies [7], we found a dose-response association between exposure to higher ACEs scores and higher odds of childhood overweight/obesity. An inverse pattern was found for PCEs; a higher number of PCEs was associated with reduced likelihood of overweight/obesity at age 8, which held true regardless of ACEs score and socioeconomic status. The observed indications for a protective effect of PCEs against childhood overweight/obesity supports findings of limited existing research [24].

In addition to protective effects, our findings also indicate that cumulative PCEs may mitigate the impact of ACEs on the development of overweight/obesity. However, at least 4 PCEs were needed for mitigating effects to manifest, and the effect of ACEs on weight status could not be alleviated when children experienced 3 or less PCEs. This is consistent with limited existing evidence suggesting that individual PCEs or a low number of PCEs is not enough to prevent or mitigate negative health outcomes [14, 20, 43].

The association between ACEs and unhealthy weight status identified in our study underscores the importance of targeting children at risk of adversity and implementing primary ACE prevention strategies. Our findings on the protective and mitigating effects of PCEs against overweight/obesity highlight the need to identify and support existing PCEs and resiliency factors within families and communities. Additionally, considering the interaction between PCEs and ACEs in relation to obesity may improve intervention efforts by leveraging existing strengths within communities and families [44].

Informed by the HOPE Framework and contrasting with highly individualized (and often victim-blaming) approaches commonly found in childhood obesity interventions, our findings on the protective and mitigating cumulative impact of PCEs underscore the importance of considering multiple ecological domains of children’s lives when conceptualizing PCEs [45].

Our findings serve as a policy, social and economic imperative for creating healthy and PCEs-promoting environments for children and their families to alleviate the burden of diseases such as obesity [23]. Through consideration of the insofar underexplored role of family environments and relationships in the prevention and treatment of childhood obesity in New Zealand, factors included in our PCE and ACE indexes can assist with the development of family-based interventions and prevention strategies. For example, our study shows that provision of appropriate and effective support for parents and families, especially those at-risk of experiencing adversity, is essential to both preventing ACEs and for mitigating their effects. Therefore, government-funded mental health and educational programs and related support services for parents should be well-funded and targeted to meet the needs of different communities. Furthermore, the importance of healthily enforced rules and routines should be emphasised for parents, healthcare workers, and educators. This is of particular importance to child obesity outcomes as explored in this study, as promotion of healthy weight behaviours may be particularly challenging if home environments are not supportive of household routines. Consistent household routines require planning, time and clear communication, and are essential to the establishment of stability and predictability for young children [46], all of which can be less frequent and more complex in families that face adversity [47].

While research has shown that early family environments and effective parenting (including responsiveness, warmth, and discipline) can influence developmental outcomes related to flourishing [44], resilience [17], and obesity [48, 49], PCEs explored in our study also highlight the need for broader evidence-based tools and intervention strategies based outside families to outweigh ACEs [44]. Directing attention towards neighbourhood and community-level contributors to obesity (such as lack of access to green/recreational spaces, social/community activities, and healthy foods) naturally aligns with ecological approaches to PCEs and ACEs research [50, 51]. Trauma-informed approaches also parallel many evidence-based strategies for supporting healthy weight behaviours in children (e.g., consistent home routines, extracurricular activities) [46, 52], which can be more complex in families who face adversity [47]. Similarly, while primary prevention of ACEs remains the best course of action [53], strategies for preventing ACEs often align with identified PCEs, such as promoting and investing in public settings like education, healthcare services, and community and recreational activities (e.g., after-school programs, libraries, pools, and social groups) [12], which may play convergent roles in supporting healthy development for children and families at the population-level. Population-level efforts must also target socioeconomic and ethnic inequities for ACEs and PCEs to ensure a more inclusive and supportive environment. Higher prevalence rates of ACEs among ethnic minority groups, particularly Māori and Pacific peoples who also face the compounded effects of racism and colonization​ [54, 55], may contribute to and further entrench inequities in obesity rates and related health outcomes. Prevention of ACEs, particularly for Māori communities, also requires addressing and healing the intergenerational and historical impacts of trauma. Trauma-informed and culturally-appropriate approaches, and adequate resourcing of such services, are especially important for communities that experience structural inequities and discrimination in access to healthcare, including obesity treatment [56, 57]. This may be of particular relevance where differences in associations between ACEs and obesity may exist, and for those with lower likelihood of experiencing PCEs.

This study supports the need for large-scale surveys and standard data collection to integrate PCEs assessment to advance knowledge of child development and wellbeing by capturing the heterogeneity of experiences across the lifecourse [20, 23], and help to identify and target areas for strengths-focused interventions and resourcing to disrupt pathways towards risks of adverse health outcomes [20, 58].

By measuring ACEs and PCEs at various time-points during childhood, our findings are less prone to recollection bias than many retrospective studies collecting data in adulthood [43, 59]. Further, obesity data was gathered using standardized objective measures, unlike previous ACEs studies that relied on parental report of child’s measurements to derive BMI [24].

### Limitations

Study attrition likely impacted our findings (Supplementary Table 3), as those who were at increased likelihood of reporting ACEs and having obesity were found to be more likely to not partake in DCW8 in which obesity data was measured. Thus, our results likely underestimated the true impact of ACEs on unhealthy weight outcomes. While the study sample remained socioeconomically and ethnically diverse, our findings should not be considered representative of the NZ children population at-large. By selecting PCEs based on sample-level indicators, our index likely had a Eurocentric bias; further research which focuses on ethnic-specific PCEs is warranted.

This study mainly relied on mother-reported data, as there were demographic differences between subjects with partner data in GUiNZ and those without. Mother-reported indicators may also have presented social desirability biases, particularly for questions related to parenting behaviours as they may invoke feelings of guilt or shame [22]. However, it was inappropriate or impractical to collect some data from children in the earlier waves. Childhood sexual abuse (which has been linked to the development of obesity) [7] had not been measured in GUiNZ at DCW 8; child-reported ACEs and PCEs should be explored as these data become available.

Our PCEs index is not a definitive concept; we focused on achievable and modifiable measures to ensure the practical application to overweight/obesity outcomes specifically, and different PCEs may have strong mitigation power to outweigh impacts of ACEs on health outcomes other than obesity, as is characteristic of research in this area [60]. Some PCE indicators collected in GUiNZ were excluded from the current study due to possible social desirability biases or lack of predictive validity for use with weight status. The breadth of PCEs considered and their testing with ACEs in individual and cumulative models will help to support further research. However, our post-hoc approach to PCEs selection may have increased the risk of type I error. While this approach offers valuable exploratory insights, it may limit the precision and replicability of the findings, particularly for outcomes other than child overweight/obesity. This study supports the need for future research to develop a standardized PCE measure tailored specifically for outcomes like child weight, to enhance consistency and generalizability.

Dichotomization of ACES and PCEs increased interpretability but may have obscured more nuanced findings. For example, “parents separated/divorced” or “mother in a committed relationship” are not infallible constructs; many children with separated parents grow up happy and healthy. These conceptual limitations are buffered by the cumulative nature of the PCEs score, echoing previous research that found that the accumulation of a sufficient number of PCEs is more important than the individual PCEs themselves [20, 61, 62]. While this study treats dichotomous PCEs cumulatively, the timing, magnitude, and consistency of PCEs could also significantly affect their impact on the development of health outcomes. Future research might explore these dimensions to identify which PCEs, when, and at what levels of intensity, offer the most effective intervention points for reducing obesity risk among children exposed to adversity. Future research should also explore clustering of individual PCEs and ACEs in cumulative models, as this may help to explain potentially spurious or inconsistent associations, such as the findings that among those with the highest number of PCEs, the highest number of ACEs (4+) was associated with lower odds of overweight/obesity than those with 1 or 2 ACEs. Sample size limitations, particularly at the ends of ranges (i.e., those with high ACEs and high PCEs or no ACEs and low PCEs) [60] may have resulted in false-negative findings in some cases.

## Conclusion

Our exploratory PCEs index contributes to a nascent field of research, particularly through the use of prospective data from a large population-based sample of children. Overall, findings suggest that presence of a sufficient number of PCEs may mitigate the detrimental impacts of ACEs on weight status among children. Our findings prompt further investigation into potential mitigating effects of PCEs on the impact of ACEs on a range of health outcomes during childhood to identify points of intervention along developmental pathways.

Significant attention must be granted to the creation, promotion, and nurturing of positive experiences that both reflect and generate resilience within children, families, and communities [20]. Provision of appropriate and effective support for parents and families, especially those at-risk for experiencing adversity, is essential to both preventing ACEs and for mitigating their effects across the life course, but also for enhancing children’s wellbeing at the population-level.

## Electronic supplementary material

Below is the link to the electronic supplementary material.

Supplementary Material 1

## Data Availability

The data utilised in the submitted manuscript was collected by the Growing Up in New Zealand study team. Researchers seeking access to this data may apply by submitting a data access application to the Data Access Committee at (dataaccess@growingup.co.nz).

## References

[1] Ward ZJ, Long MW, Resch SC, Giles CM, Cradock AL, Gortmaker SL. Simulation of Growth trajectories of Childhood obesity into Adulthood. N Engl J Med. 2017;377(22):2145–53. 10.1056/NEJMoa1703860. 29171811 10.1056/NEJMoa1703860 PMC9036858

[2] Craigie AM, Matthews JNS, Rugg-Gunn AJ, Lake AA, Mathers JC, Adamson AJ. Raised adolescent body Mass Index predicts the development of Adiposity and a central distribution of body Fat in Adulthood: a longitudinal study. Obes Facts. 2009;2(3):150–6. 10.1159/000218092. 20054219 10.1159/000218092 PMC6516202

[3] Daniels SR. The consequences of Childhood overweight and obesity. Future Child. 2006;16(1):47–67. 10.1353/foc.2006.0004. 16532658 10.1353/foc.2006.0004

[4] Sahoo K, Sahoo B, Choudhury AK, Sofi NY, Kumar R, Bhadoria AS. Childhood obesity: causes and consequences. *Journal of family medicine and primary care*. Apr-Jun. 2015;4(2):187–92. 10.4103/2249-4863.154628. 10.4103/2249-4863.154628 PMC4408699 25949965

[5] Abarca Gómez L, Sunyer Deu J, Vrijheid M, Ezzati M, Collaboration NCDRF. Worldwide trends in body-mass index, underweight, overweight, and obesity from 1975 to 2016: a pooled analysis of 2416 population-based measurement studies in 128·9 million children, adolescents, and adults. 2017.10.1016/S0140-6736(17)32129-3 10.1016/S0140-6736(17)32129-3 PMC5735219 29029897

[6] Baranowski T, Motil KJ, Moreno JP. Behavioral Research Agenda in a Multietiological Approach to child obesity Prevention. Child Obes. 2019;15(4):223–6. 10.1089/chi.2019.0052. 30925082 10.1089/chi.2019.0052 PMC6622575

[7] Schroeder K, Schuler BR, Kobulsky JM, Sarwer DB. The association between adverse childhood experiences and childhood obesity: a systematic review. Obes Rev. 2021;22(7):e13204–n/a. 10.1111/obr.13204. 33506595 10.1111/obr.13204 PMC8192341

[8] Hemmingsson E. New model of the role of psychological and emotional distress in promoting obesity: conceptual review with implications for treatment and prevention. Obes Rev. 2014;15(9):769–79. 10.1111/obr.12197. 24931366 10.1111/obr.12197

[9] Heerman WJ, Krishnaswami S, Barkin SL, McPheeters M. Adverse family experiences during childhood and adolescent obesity. Obes (Silver Spring) Mar. 2016;24(3):696–702. 10.1002/oby.21413. 10.1002/oby.21413 PMC4769653 26853526

[10] Felitti VJ, Anda RF, Nordenberg D, et al. Relationship of childhood abuse and household dysfunction to many of the leading causes of death in adults: the adverse childhood experiences (ACE) study. Am J Prev Med. 1998;14(4):245–58. 10.1016/S0749-3797(98)00017-8. 9635069 10.1016/s0749-3797(98)00017-8

[11] Brown DWDMM, Anda RFMDM, Tiemeier HP, et al. Adverse childhood experiences and the risk of premature mortality. Am J Prev Med. 2009;37(5):389–96. 10.1016/j.amepre.2009.06.021. 19840693 10.1016/j.amepre.2009.06.021

[12] Centers for Disease Control and Prevention. About the CDC-Kaiser ACE study. Atlanta, GA. https://www.cdc.gov/violenceprevention/aces/about.html

[13] Bethell CD, Newacheck P, Hawes E, Halfon N. Adverse childhood experiences: Assessing the impact on health and school engagement and the mitigating role of resilience. *Health Affairs*. Dec 2014 2021-09-09 2014;33(12):2106–2115. 10.1377/hlthaff.2014.0914 10.1377/hlthaff.2014.0914 25489028

[14] Crandall A, Broadbent E, Stanfill M, et al. The influence of adverse and advantageous childhood experiences during adolescence on young adult health. Child Abuse Negl. 2020;108:104644–104644. 10.1016/j.chiabu.2020.104644. 32795716 10.1016/j.chiabu.2020.104644

[15] Sege RD, Harper Browne C. Responding to ACEs with HOPE: Health outcomes from positive experiences. Acad Pediatr. 2017;17(7):S79–85. 10.1016/j.acap.2017.03.007. 28865664 10.1016/j.acap.2017.03.007

[16] Crouch E, Smith HP, Andersen TS. An examination of caregiver incarceration, positive childhood experiences, and school success. Child Youth Serv Rev. 2022;133:106345. 10.1016/j.childyouth.2021.106345.

[17] Liu SR, Kia-Keating M, Nylund-Gibson K, Barnett ML. Co-occurring Youth profiles of adverse childhood experiences and protective factors: associations with Health, Resilience, and racial disparities. Am J Community Psychol. 2020;65(1–2):173–86. 10.1002/ajcp.12387. 31489651 10.1002/ajcp.12387

[18] Bachler E, Frühmann A, Bachler H, Aas B, Nickel M, Schiepek GK. The Effect of Childhood adversities and protective factors on the Development of Child-Psychiatric disorders and their treatment. Front Psychol. 2018. 10.3389/fpsyg.2018.02226. 30524336 10.3389/fpsyg.2018.02226 PMC6262315

[19] Yule K, Houston J, Grych J. Resilience in children exposed to violence: a Meta-analysis of protective factors across ecological contexts. Clin Child Fam Psychol Rev. 2019;22(3):406–31. 10.1007/s10567-019-00293-1. 30887404 10.1007/s10567-019-00293-1

[20] Bethell C, Jones J, Gombojav N, Linkenbach J, Sege R. Positive Childhood Experiences and Adult Mental and Relational Health in a Statewide Sample: associations across adverse childhood experiences levels. JAMA Pediatr. 2019;173(11):e193007. 10.1001/jamapediatrics.2019.3007. 31498386 PMC6735495

[21] Narayan AJ, Rivera LM, Bernstein RE, Harris WW, Lieberman AF. Positive childhood experiences predict less psychopathology and stress in pregnant women with childhood adversity: a pilot study of the benevolent childhood experiences (BCEs) scale. Child Abuse Negl. 2018;78:19–30. 10.1016/j.chiabu.2017.09.022. 28992958 10.1016/j.chiabu.2017.09.022

[22] Guo S, O’Connor M, Mensah F, et al. Measuring positive childhood experiences: testing the structural and predictive validity of the Health outcomes from positive experiences (HOPE) Framework. Acad Pediatr. 2021;22(6):942–51. 10.1016/j.acap.2021.11.003. 34801761 10.1016/j.acap.2021.11.003

[23] Merrick JS, Narayan AJ. Assessment and screening of positive childhood experiences along with childhood adversity in research, practice, and policy. J Child Poverty. 2020;26(2):269–81. 10.1080/10796126.2020.1799338.

[24] Crouch E, Radcliff E, Kelly K, Merrell MA, Bennett KJ. Examining the influence of positive childhood experiences on childhood overweight and obesity using a national sample. Prev Med. 2022;154106907. 10.1016/j.ypmed.2021.106907. 10.1016/j.ypmed.2021.106907 34864065

[25] Delany DE, Cheung CS. Culture and Adolescent Development. *The encyclopedia of child and adolescent development*. 2020:1–12.

[26] Brown JD, Dutton KA, Cook KE. From the top down: Self-esteem and self-evaluation. *Cognition and Emotion*. 2001/09/01 2001;15(5):615–631. 10.1080/02699930126063

[27] Lopes PN, Salovey P, Coté S, Beers M. Emotion regulation abilities and the quality of social interaction. Emot Mar. 2005;5(1):113–8. 10.1037/1528-3542.5.1.113. 10.1037/1528-3542.5.1.113 15755224

[28] Fritz J, de Graaff A, Caisley H, van Harmelen A-L, Wilkinson PO. A systematic review of amenable resilience factors that moderate and/or mediate the Relationship between Childhood Adversity and Mental Health in Young people. Front Psychiatry. 2018;9(June):1–17. 10.3389/fpsyt.2018.00230. 29971021 10.3389/fpsyt.2018.00230 PMC6018532

[29] Afifi TO, MacMillan HL. Resilience following child maltreatment: a review of protective factors. Can J Psychiatry. 2011;56(5):266–72. 10.1177/070674371105600505. 21586192 10.1177/070674371105600505

[30] Rasmussen LJH, Moffitt TE, Arseneault L, et al. Association of Adverse Experiences and exposure to violence in Childhood and Adolescence with Inflammatory Burden in Young people. JAMA Pediatr. 2020;174(1):38–47. 10.1001/jamapediatrics.2019.3875. 10.1001/jamapediatrics.2019.3875 PMC6830440 31682707

[31] Danese A, Baldwin JR. Hidden wounds? Inflammatory links between Childhood Trauma and Psychopathology. Annual Rev Psychol 2017/01/03. 2017;68(1):517–44. 10.1146/annurev-psych-010416-044208. 10.1146/annurev-psych-010416-044208 27575032

[32] Slopen N, Chen Y, Priest N, Albert MA, Williams DR. Emotional and instrumental support during childhood and biological dysregulation in midlife. *Preventive Medicine*. 2016/03/01/ 2016;84:90–96. doi:https://doi.org/10.1016/j.ypmed.2015.12.003. 10.1016/j.ypmed.2015.12.003 PMC4758867 26708307

[33] Priest N, Guo S, Gondek D et al. The effect of adverse and positive experiences on inflammatory markers in Australian and UK children. Brain, Behavior, & Immunity - Health. 2022/12/01/ 2022;26:100550. 10.1016/j.bbih.2022.100550 10.1016/j.bbih.2022.100550 PMC9677086 36420372

[34] O’Brien JR, Loi EC, Byrne ML, Zalewski M, Casement MD. The Link between positive and negative parenting behaviors and child inflammation: a systematic review. Child Psychiatry Hum Dev Feb. 2023;54(1):51–65. 10.1007/s10578-021-01224-4. 10.1007/s10578-021-01224-4 PMC8814056 34347228

[35] Heerman WJ, Samuels LR, González Peña T, et al. Family resilience and childhood obesity among children exposed to adverse childhood experiences in a national survey. Obes Sci Pract. 2022;8(1):3–11. 10.1002/osp4.497. 35127118 10.1002/osp4.497 PMC8804940

[36] Morton SMB, Ramke J, Kinloch J, et al. Growing up in New Zealand cohort alignment with all New Zealand births. Aust N Z J Public Health. 2015;39(1):82–7. 10.1111/1753-6405.12220. 25168836 10.1111/1753-6405.12220

[37] von Elm E, Altman DG, Egger M, Pocock SJ, Gøtzsche PC, Vandenbroucke JP. The strengthening the reporting of Observational studies in Epidemiology (STROBE) Statement: guidelines for reporting observational studies. Epidemiology. 2007;18(6):800–4. 10.1097/EDE.0b013e3181577654. 18049194 10.1097/EDE.0b013e3181577654

[38] Crouch E, Radcliff E, Merrell MA, Bennett KJ. Rural-urban differences in Positive Childhood Experiences across a National Sample. J Rural Health. 2021;37(3):495–503. 10.1111/jrh.12493. 32639648 10.1111/jrh.12493

[39] Atkinson J, Salmond C, Crampton PJWDPH. University of Otago. NZDep2013 index of deprivation. 2014.

[40] *Stata Statistical Software: release 15*. StataCorp LLC; 2017.

[41] Hashemi L, Fanslow J, Gulliver P, McIntosh T. Exploring the health burden of cumulative and specific adverse childhood experiences in New Zealand: Results from a population-based study. Child Abuse & Neglect. 2021;122 Article 105372. 10.1016/j.chiabu.2021.105372 10.1016/j.chiabu.2021.105372 34717153

[42] Houtepen LC, Heron J, Suderman MJ, Fraser A, Chittleborough CR, Howe LD. Associations of adverse childhood experiences with educational attainment and adolescent health and the role of family and socioeconomic factors: a prospective cohort study in the UK. PLoS Med. 2020;17(3):e1003031. 10.1371/journal.pmed.1003031. 32119668 10.1371/journal.pmed.1003031 PMC7051040

[43] Bellis MA, Hughes K, Ford K, et al. Adverse childhood experiences and sources of childhood resilience: a retrospective study of their combined relationships with child health and educational attendance. BMC Public Health. 2018;18(1):792–792. 10.1186/s12889-018-5699-8. 29940920 10.1186/s12889-018-5699-8 PMC6020215

[44] Bethell CD, Gombojav N, Whitaker RC. Family resilience and connection promote flourishing among US children, even amid adversity. Health Aff. 2019/05/01 2019;38(5):729–37. 10.1377/hlthaff.2018.05425 10.1377/hlthaff.2018.05425 31059374

[45] Cicchetti D, Toth SL. Child maltreatment and developmental psychopathology: A multilevel perspective. *Developmental psychopathology: Maladaptation and psychopathology, Vol 3, 3rd ed*. John Wiley & Sons, Inc.; 2016:457–512.

[46] Fiese BH, Rhodes HG, Beardslee WR. Rapid changes in American family life: consequences for child health and pediatric practice. Pediatr Sep. 2013;132(3):552–9. 10.1542/peds.2013-0349. 10.1542/peds.2013-0349 PMC3876759 23918891

[47] Evans GW, Gonnella C, Marcynyszyn LA, Gentile L, Salpekar N. The role of chaos in poverty and children’s socioemotional adjustment. Psychol Sci Jul. 2005;16(7):560–5. 10.1111/j.0956-7976.2005.01575.x. 10.1111/j.0956-7976.2005.01575.x 16008790

[48] Schrempft S, van Jaarsveld CHM, Fisher A, et al. Variation in the heritability of child body Mass Index by Obesogenic Home Environment. JAMA Pediatr. 2018;172(12):1153–60. 10.1001/jamapediatrics.2018.1508. JAMA Pediatrics. PMC6396810 30285028

[49] Brotman LM, Dawson-McClure S, Huang K-Y, et al. Early Childhood Family intervention and long-term obesity Prevention among High-risk Minority Youth. Pediatrics. 2012;129(3):e621–8. 10.1542/peds.2011-1568. 10.1542/peds.2011-1568 PMC3289522 22311988

[50] Schroeder K, Schuler BR, Kobulsky JM, Sarwer DB. The association between adverse childhood experiences and childhood obesity: a systematic review. Obes Rev Jul. 2021;22(7):e13204. 10.1111/obr.13204. 10.1111/obr.13204 PMC8192341 33506595

[51] Carrillo-Álvarez E, Kawachi I, Riera-Romaní J. Neighbourhood social capital and obesity: a systematic review of the literature. Obes Rev Jan. 2019;20(1):119–41. 10.1111/obr.12760. 10.1111/obr.12760 30306717

[52] Teevale T, Kaholokula JK. Using appreciative inquiry methodology to develop a weight management program for obese children in New Zealand. Aust N Z J Public Health Feb. 2018;42(1):7–11. 10.1111/1753-6405.12719. 10.1111/1753-6405.12719 28898503

[53] Shonkoff JP. Capitalizing on Advances in Science to Reduce the Health Consequences of Early Childhood Adversity. JAMA Pediatr. 2016;170(10):1003–7. 10.1001/jamapediatrics.2016.1559. JAMA Pediatrics. 27548291

[54] Joy E, Beddoe L. ACEs, cultural considerations and ‘common sense’in Aotearoa New Zealand. Social Policy Soc. 2019;18(3):491–7.

[55] Pihama L, Reynolds P, Smith C, Reid J, Smith LT, Nana RT. Positioning historical trauma theory within Aotearoa New Zealand. AlterNative: Int J Indigenous Peoples. 2014;2014/09(01):248–62. 10.1177/117718011401000304.

[56] Pihama L, Smith LT, Evans-Campbell T et al. Investigating Māori approaches to trauma informed care. 2017.

[57] Pihama L, Cameron N, Nana RT. *Historical trauma and whānau violence*. 2019. https://apo.org.au/node/263601

[58] Larkin H, Felitti VJ, Anda RF. Social work and adverse childhood experiences Research: implications for practice and Health Policy. Social work Public Health. 2014;29(1):1–16. 10.1080/19371918.2011.619433. 10.1080/19371918.2011.619433 24188292

[59] Bellis MA, Hardcastle K, Ford K, et al. Does continuous trusted adult support in childhood impart life-course resilience against adverse childhood experiences - a retrospective study on adult health-harming behaviours and mental well-being. BMC Psychiatry. 2017;17(1):110–110. 10.1186/s12888-017-1260-z. 28335746 10.1186/s12888-017-1260-z PMC5364707

[60] Luthar SS, Cicchetti D, Becker B. The construct of resilience: a critical evaluation and guidelines for future work. Child Dev. 2000;71(3):543–62. 10.1111/1467-8624.00164. 10953923 10.1111/1467-8624.00164 PMC1885202

[61] Xu Z, Zhang D, Ding H, et al. Association of positive and adverse childhood experiences with risky behaviours and mental health indicators among Chinese university students in Hong Kong: an exploratory study. Eur J Psychotraumatol. 2022;13(1):2065429. 10.1080/20008198.2022.2065429. 35646294 10.1080/20008198.2022.2065429 PMC9135422

[62] Crandall A, Miller JR, Cheung A, et al. ACEs and counter-ACEs: how positive and negative childhood experiences influence adult health. Child Abuse Negl. 2019;96104089. 10.1016/j.chiabu.2019.104089. 10.1016/j.chiabu.2019.104089 31362100

[63] Johnson MP, Caughlin JP, Huston TL. The tripartite nature of marital commitment: Personal, moral, and structural reasons to stay married. J Marriage Fam. 1999;61(1):160–77. 10.2307/353891.

[64] Aram D, Levin I. Mother–child joint writing in low SES: sociocultural factors, maternal mediation, and emergent literacy. Cogn Dev. 2001;16(3):831–52. 10.1016/S0885-2014(01)00067-3.

[65] Aram D, Levin I. The role of maternal mediation of writing to kindergartners in promoting literacy in school: a longitudinal perspective. Read Writ. 2004;17(4):387–409. 10.1023/B:READ.0000032665.14437.e0.

[66] Growing Up In New Zealand. External Data Release 2020 Data User Guide. 2020. https://www.growingup.co.nz/available-data-2

[67] Mahoney JL, Magnusson D. Parent participation in community activities and the persistence of criminality. Dev Psychopathol. 2001;13(1):125–41. 10.1017/S0954579401001092. 11346047 10.1017/s0954579401001092

[68] Traub F, Boynton-Jarrett R. Modifiable resilience factors to Childhood Adversity for Clinical Pediatric Practice. Pediatrics. 2017;139(5):e20162569. 10.1542/peds.2016-2569. 28557726 10.1542/peds.2016-2569

[69] Larose MP, Côté SM, Ouellet-Morin I, Maughan B, Barker ED. Promoting better functioning among children exposed to high levels of family adversity: the protective role of childcare attendance. J Child Psychol Psychiatry. 2021;62(6):762–70. 10.1111/jcpp.13313. 32827178 10.1111/jcpp.13313

[70] Sciaraffa MA, Zeanah PD, Zeanah CH. Understanding and promoting resilience in the context of adverse childhood experiences. Early Childhood Educ J. 2018;46(3):343–53. 10.1007/s10643-017-0869-3.

[71] Cormack D, Robson C. Ethnicity, national identity and’New Zealanders’: Considerations for monitoring Māori health and ethnic inequalities. Rōpū Rangahau Hauora a Eru Pōmare; 2010.

