# Supplementary material for: Identification of positive childhood experiences with the potential to mitigate childhood unhealthy weight status in children within the context of adverse childhood experiences: a prospective cohort study
Source: BMC Public Health. 2025 Jan 13;25:8. doi: 10.1186/s12889-024-20727-y (PMC11727318; doi:10.1186/s12889-024-20727-y)
Supplement: Supplementary file 1 — Supplementary Material 1 [file 12889_2024_20727_MOESM1_ESM.docx]

**Supplementary Table 1.** Definitions of Adverse Childhood Experiences used in analysis of Growing Up in New Zealand study

| Individual ACE | Description |
| --- | --- |
| Emotional abuse | A child was classified as being emotionally abused if mothers and/or partner reported the following in DCW2, DCW54M, or DCW8:  - **Always or almost always** have lost their temper with child, raised their voice, yelled or shouted at child (mother reported, DCW8),  - **Very often or always** yelled or shouted when child misbehaved or exploded with anger (mother reported,DCW54M),  - **Very often** shouted when child was naughty or **very often, extremely often, or all the time** got angry at the child, criticize their child’s ideas, shouted at the child because they were upset with their child (mother and/or partner reported, DCW2). |
| Physical abuse | Children were coded as having this ACE if:   - Mother responded **‘often’, ‘always or almost always’** to using physical punishment such as smacking as a way of interacting with their child (DCW8) - Mother used physical punishment as a way of disciplining their child ‘**half the time’, ‘very often’, or ‘always’** (DCW54M). - Mother reported smacking the child **‘often’ ‘very often’** when he/she was naughty (DCW2). |
| Parental substance abuse (including alcohol abuse) | A child was coded as exposed to this ACE if the mother or partner reported any of the followings:   - Mother reported that the child has experienced this ACE (drug taking / alcoholism in the immediate family, DCW8) - Mother reported heavy episode drinking (drinking 5 or more standard drinks on a typical day when drinking, DCW54M, DCW8, or drinking 6 or more standard drinks on one occasion at least monthly, DCW54M and DCW8). - Mother reported drinking 10 or more drinks per week (DCW54M) - Mother ever sought help in relation to alcohol use (DCW54M) - Mother or partner reported using amphetamines, cocaine, ecstasy, opiates, hallucinogens or party pills since the birth of the child (DCW9M) |
| Parental mental illness | A child was assigned to this ACE if any of the following was the case:   - Mother reported that the child has been exposed to mental illness in the immediate family (DCW8) - Mother or partner was classified as moderately or severely depressed (a score of 15 or higher on the Patient Health Questionnaire Depression Screener) or as probably depressed (a score of 12 or higher onEdinburgh Postnatal Depression Scale (DCW8, DCW54M, DCW9M, or DCW0) |
| Parental incarceration | A child was assigned to this ACE if any of the following was the case:   - Mother reported that the child has experienced this ACE (parent in prison, DCW8) - Mother reported that she has ever been convicted of a crime which resulted in a jail sentence (DCW54M) - Partner reported that he has ever been convicted of a crime which resulted in a jail sentence (DCW9M) |
| Parental separation/ divorce | Child was coded as having this ACE if the mother reported any of the following:   - Child has experienced divorce/ separation of parents (DCW8) - Mother was not in a relationship with this same partner when her child was two years old - Mother did not have a current partner during 54months, 2 years, or 9 months interviews. |
| Intimate partner violence (IPV) | A child was coded as having this ACE if mother reported:   - Child was present when she had a physical conflict with her partner. - Or, mother reported any of the following during past 4 weeks prior to the data collection in any frequency other than never: - Their partner slapped them or threw things at them that could have hurt them; pushed or shoved them or pulled their hair; hit them with a fist or something else that could have hurt them (DCW54M, DCW8) or - Mother and partner pushed and shoved each other while arguing (DCW9M) |
| Mother discrimination | A child was coded as having this ACE if mother reported that:   - She has ever been treated unfairly in New Zealand because of her ethnicity (DCW2)   Or mother has ever felt any of the following within the past 12 months or more than 12 months ago (DCW0):   - Being a victim of an ethnically motivated attack - that is verbal, or physical abuse to the person or property in New Zealand - Being treated unfairly (e.g., treated differently, kept waiting) by a health professional, e.g., a doctor, nurse, dentist because of your ethnicity in New Zealand - Being treated unfairly at work or been refused a job because of your ethnicity in New Zealand - Being treated unfairly when renting or buying housing because of your ethnicity in New Zealand - Being treated unfairly by the police, the justice system (courts), or the corrections department (prison, community service, periodic detention, parole, probation) because of your ethnicity in New Zealand - Being treated unfairly when asking for loans, a mortgage, hire purchase or credit cards because of your ethnicity in New Zealand - Being treated unfairly when attending a place of learning because of your ethnicity in New Zealand |
| Recurrent peer-bullying/peer victimisation | A child was coded as having this ACE if he/she reported frequent experience of one of the following (at least 1 or 2 times a month):   - Do other children put you down, call you names, or tease you in a mean way? - Do other children leave you out in a mean way? - Do other students hit, push, or hurt you in a mean way? - Do other children tell lies about you in a mean way? - Do other children threaten you in a mean way, or force you to do things? - Do other children take or break your stuff in a mean way (e.g., money or pens)? - Do other children say mean things about your culture or family? - Are other children mean to you because you learn in a different way to them? - Do other children use cell phones (like texting) or the Internet (like Facebook) to be mean to? - At school, are you bullied by other students? |

Note: DCW = Data Collection Wave. Data taken from DCW8Y includes items drawn from ACEs list as well as items taken from other parts of this DCW.

**Supplementary Table 2.**Multivariable models of odds of overweight/obesity by ACEs scores adjusted for covariates and individual PCEs, and individual PCEs/ACEs interaction terms adjusted for ACEs scores and covariates

|  | Overweight/obesity  OR/AOR [95%CI] | | | | | | | |
| --- | --- | --- | --- | --- | --- | --- | --- | --- |
|  | Model 0: Unadjusted for PCE | | Model 1: Mother in committed relationship | Model 2: Mother interacted well with child | Model 3: Mother involved in social groups | Model 4: Child engaged in activities | Model 5: Child lived in home with routines & rules | Model 6: Mother was satisfied with ECE |
| ACEs score (Ref=0) | OR | AOR^a^ | AOR^b^ | | | | | |
| *1 ACE* | **1.41 [1.13-1.76]** | **1.45 [1.14-1.84]** | **1.47 [1.16-1.87]** | **1.42 [1.11-1.81]** | **1.48 [1.17-1.88]** | **1.44 [1.14-1.83]** | **1.43 [1.13-1.82]** | **1.44 [1.13-1.83]** |
| *2 ACEs* | **1.72 [1.38-2.14]** | **1.51 [1.19-1.92]** | **1.51 [1.19-1.93]** | **1.48 [1.15-1.89]** | **1.52 [1.20-1.94]** | **1.51 [1.19-1.92]** | **1.48 [1.17-1.88]** | **1.51 [1.18-1.92]** |
| *3 ACEs* | **2.34 [1.85-2.95]** | **1.84 [1.43-2.38]** | **1.85 [1.42-2.40]** | **1.83 [1.40-2.38]** | **1.86 [1.44-2.42]** | **1.86 [1.44-2.41]** | **1.78 [1.37-2.30]** | **1.81 [1.39-2.34]** |
| *4+ ACEs* | **3.58 [2.83-4.52]** | **2.30 [1.77-3.00]** | **2.29 [1.74-3.00]** | **2.21 [1.69-2.91]** | **2.32 [1.78-3.03]** | **2.27 [1.74-2.96]** | **2.20 [1.69-2.87]** | **2.34 [1.79-3.06]** |
| Interaction: PCE x ACEs | | | | | | | | |
| *PCE x 1 ACE* | | | 1.03 [0.54-1.97] | 0.60 [0.35-1.02] | 0.88 [0.50-1.55] | **0.59 [0.36-0.97]** | 0.93 [0.58-1.50] | 0.87 [0.54-1.42] |
| *PCE x 2 ACEs* | | | 0.86 [0.45-1.62] | 0.88 [0.52-1.50] | 0.83 [0.48-1.46] | 0.73 [0.45-1.19] | 0.77 [0.48-1.25] | 0.82 [0.50-1.33] |
| *PCE x 3 ACEs* | | | 1.24 [0.64-2.38] | 0.62 [0.36-1.09] | 0.97 [0.54-1.73] | 0.72 [0.42-1.21] | 1.22 [0.72-2.05] | 0.73 [0.43-1.22] |
| *PCE x 4+ ACEs* | | | 0.98 [0.51-1.89] | **0.52 [0.30-0.92]** | 0.93 [0.52-1.64] | 0.77 [0.46-1.30] | 0.75 [0.44-1.27] | 0.61 [0.36-1.03] |

**^a^**AOR in model 0 is adjusted for child’s prioritized ethnicity (DCW4), child’s gender (DCW0), food insecurity (DCW8). **^b^**AORs in models 1-6 are adjusted for covariates in Model 0 and for that particular individual PCE

**Supplementary Table 3:** Sociodemographic characteristics of participants with obesity data at DCW8 compared to non-participants in DCW8

|  | No. (Col %) | | Unadjusted odds ratio [95%CI] | |
| --- | --- | --- | --- | --- |
|  | **Children without obesity data at DCW8** | **Children with obesity data at DCW8** | **Missing obesity data DCW8** | **Overweight/obesity at 8Y** |
|  | 1,862 (27.56) | 4,895 (72.44) |  |  |
| **Child’s gender (DCW1)** | | | | |
| Boy | 968 (51.99) | 2,522 (51.52) | Ref. | Ref. |
| Girl | 894 (48.01) | 2,373 (48.48) | 0.98 [0.88-1.09] | 0.90 [0.80-1.02] |
| **Mother’s ethnicity (DCW0)** | | | | |
| Māori | 481 (25.83) | 764 (15.61) | **3.85 [3.31-4.47]** | **2.77 [2.35-3.27]** |
| Pacific | 506 (27.18) | 497 (10.15) | **6.22 [5.31-7.28]** | **7.67 [6.19-9.51]** |
| Asian | 343 (18.42) | 687 (14.03) | **3.05 [2.59-3.59]** | 0.98 [0.81-1.18] |
| MELAA | 61 (3.28) | 90 (1.84) | **4.14 [2.95-5.81]** | 1.29 [0.82-2.03] |
| European/NZer | 467 (25.08) | 2,853 (58.28) | Ref. | Ref. |
| Missing | <10 (0.21) | <10 (0.08) |  |  |
| **Mother’s age group at pregnancy (DCW0)** | | | | |
| Less than 20 years | 157 (8.43) | 168 (3.43) | **3.51 [2.76-4.47]** | **2.11 [1.53-2.90]** |
| 20-24 years | 420 (22.56) | 564 (11.52) | **2.80 [2.37-3.30]** | **2.02 [1.66-2.45]** |
| 25-29 years | 495 (26.58) | 1,153 (23.55) | **1.61 [1.39-1.87]** | **1.40 [1.19-1.64]** |
| 30-34 years | 442 (23.74) | 1,660 (33.91) | Ref. | Ref. |
| 35-39 years | 282 (15.15) | 1,129 (23.06) | 0.94 [0.79-1.11] | 1.10 [0.94-1.30] |
| 40+ years | 66 (3.54) | 221 (4.51) | 1.12 [0.84-1.51] | 1.03 [0.76-1.40] |
| **Mother’s education (DCW0)** | | | | |
| No secondary school qualification | 239 (12.84) | 239 (4.88) | **5.04 [4.03-6.31]** | **3.35 [2.52-4.44]** |
| Secondary school qualification | 568 (30.50) | 1,039 (21.23) | **2.76 [2.33-3.26]** | **1.76 [1.48-2.09]** |
| Trade certificate/diploma | 607 (32.60) | 1,460 (29.83) | **2.10 [1.78-2.47]** | **1.69 [1.44-1.99]** |
| Bachelor’s degree | 253 (13.59) | 1,275 (26.05) | Ref. | Ref. |
| Higher degree | 185 (9.94) | 873 (17.83) | 1.07 [0.87-1.32] | 0.79 [0.65-0.97] |
| Missing | 10 (0.54) | <10 (0.18) |  |  |
| **Mother in relationship (DCW0)** | | | | |
| Yes | 1,516 (81.42) | 4,262 (87.07) | Ref. | Ref. |
| No | 133 (7.14) | 193 (3.94) | **1.94 [1.54-2.43]** | **1.82 [1.36-2.43]** |
| Missing | 213 (11.44) | 440 (8.99) |  |  |
| **Mother born in NZ (DCW0)** | | | | |
| Yes | 1,004 (53.92) | 3,333 (68.09) | Ref. | Ref. |
| No | 855 (45.92) | 1,558 (31.83) | **1.82 [1.63-2.03]** | 0.91 [0.80-1.03] |
| Missing | <10 (0.16) | <10 (0.08) |  |  |
| **Area deprivation level (DCW0)** | | | | |
| Least deprived (NZDep 1-3) | 296 (15.90) | 1,388 (28.36) | Ref. | Ref. |
| Moderately deprived (NZDep 4-7) | 528 (28.36) | 1,941 (39.65) | **1.28 [1.09-1.49]** | 1.09 [0.94-1.27] |
| Most deprived (NZDep 8-10) | 1,037 (55.69) | 1,565 (31.97) | **3.11 [2.68-3.60]** | **2.42 [2.07-2.82]** |
| Missing | <10 (0.05) | <10 (0.02) |  |  |
| **Parental incarceration (DCW9M, 54M, 8Y)** | | | | |
| No | 1391 (74.70) | 4774 (97.5) | Ref. | Ref. |
| Yes | 46 (2.47) | 102 (2.08) | **1.55 [1.09-2.20]** | **1.59 [1.07-2.36]** |
| Missing | 425 (22.82) | 19 (0.39) |  |  |
| **IPV against mother (DCW9M, 54M, 8Y)** | | | | |
| No | 1206 (64.77) | 4180 (85.39) | Ref. | Ref. |
| Yes | 288 (15.47) | 537 (10.97) | **1.86 [1.59-2.17]** | 1.76 [1.47-2.11] |
| Missing | 368 (19.76) | 178 (3.64) |  |  |
| **Overweight/obesity (DCW4.5)** | | | | |
| Thinness/normal | 742 (39.85) | 4,142 (84.62) | Ref. | Ref. |
| Overweight/obesity | 201 (10.79) | 545 (11.13) | 2.06 [1.72-2.46] | 10.88 [8.70-13.60] |
| Missing (incl. outliers) | 919 (49.36) | 208 (4.25) |  |  |

^a^ Cells have fewer than 10 cases. Exact numbers not reported to protect the anonymity of participant
DCW = Data Collection Wave
